# Gender gap in citations, h-index, and representation: Examining the highly cited authors across continents and disciplines in Google Scholar

**DOI:** 10.1371/journal.pone.0334690

**Published:** 2025-11-13

**Authors:** Manuel Goyanes, Adrián Domínguez-Díaz, Beatriz Jordá, Luis de-Marcos

**Affiliations:** 1 Department of Communication, MITCOM Research Group, Universidad Carlos III de Madrid, Spain; 2 Department of Computer Science, Universidad de Alcalá, Spain; 3 Department of English, Saint Louis University, Madrid campus, Spain; KTH Royal Institute of Technology, SWEDEN

## Abstract

This study investigates gender inequalities in academia by examining differences in representation, citations, and h-index between male and female highly cited researchers across disciplines and geographic regions. Using a unique dataset from Google Scholar, this study analyzes 21,509 highly cited authors across 191 fields and all continents. We examine gender disparities in citations, h-index, and representation while controlling for research productivity and career length to determine if female researchers experience different outcomes compared to their male counterparts. The findings reveal that women are significantly underrepresented among highly cited scholars globally (0.255 women per man) and receive fewer citations and have lower h-indexes than men in most regions and disciplines. However, after controlling for productivity and career length, female scholars are cited more than men in the pooled sample, Asia, Europe, and in two fields (natural sciences and exact sciences/physics). Despite this, women’s h-index remains significantly lower than men’s in all regions except Africa and South America, and in all fields except social sciences. This study highlights the persistence of gender inequalities in academic representation and long-term impact, as measured by the h-index. The results suggest that while citation rates for female researchers can match or exceed those of male scholars when productivity is controlled for, structural barriers continue to limit women’s long-term recognition in academia. This research contributes to the understanding of gender disparities among top researchers, showing that while citation parity is possible, significant gender gaps remain in overall academic representation and long-term recognition through h-index measures.

The inequality between women and men in science continues to be prevalent despite signs of improvement in recent decades [1]. Extant research has evidenced that, compared to men, women are less cited and productive across fields [2–5], publish less often in prestigious journals [6,7], win fewer important research grants [8], are less represented on the editorial boards of top-tier journals [9], and occupy less often leadership or seniority positions in terms of authorship and research projects [7,10,11]. The gap in research impact, in particular, is a crucial aspect that has received a lot of attention in the literature, as it is an important indicator of gender differences in academic success and influence [12,13].

This study expands this body of research by examining the gender gap in representation, citations, and long-term impact (i.e., h-index) among highly cited researchers across all disciplines and continents using data from Google Scholar. We focus on this group of scholars because they play a crucial role in science: they are far more prolific than other researchers and exert considerable influence in their disciplines, often pioneering new avenues of research or publishing the majority of top-cited papers [14,15]. Previous studies have examined top researchers’ differences by looking at specific countries or fields, or have neglected important factors like individual productivity [13,16–18]. This has complicated cross-comparisons across geographical areas and disciplines, making it difficult to generalize findings to the entire scientific community. In addition, most studies relied mainly on data from large-scale platforms such as SciVal or Web of Science, typically neglecting Google Scholar, despite it being one of the most popular academic search engines and increasingly relevant for bibliographic research [19,20].

Our findings, based on a database of 21,509 authors from 194 scientific fields gathered from Google Scholar, show that female researchers are significantly underrepresented across continents and fields, and that they obtain fewer citations and have a lower h-index across all fields and continents, except for South America (in both citations and h-index) and Africa (in h-index). Moreover, results show that when considering research productivity and experience, female scholars are significantly more cited than males in Asia and Europe and two fields (natural sciences, and exact sciences and physics). The remaining fields show no statistically significant differences. However, female scholars have a significantly lower h-index in all geographical regions except Africa and South America, and in all scientific fields except social sciences. Overall, our study provides the literature with a comprehensive picture of the existing gender disparities among highly cited scholars. It also underlines the multifaceted nature of the gender gap in science and the need for continued research and targeted interventions to address these challenges.

## The gender gap in science

In recent decades, the presence of women in higher education and science has substantially increased. Globally, women constituted nearly 44% of university professors in 2020, a significant increase from less than 35% in 1990 [1]. However, although female researchers have improved their positions, several studies have noted that important gender differences still persist in science regarding impact, research productivity, collaboration networks, and salary [2,18,21,22]. Furthermore, in many countries, the presence of women gradually declines as seniority levels increase, with only a small number achieving senior or leadership positions within higher education institutions [1,17].

One of the most scrutinized issues in this body of research is the gender gap in citations. Even with varying methods, control variables, databases, scientific fields, and countries, most studies consistently find that women generally get cited less often than men [2,4,12,22], with a few exceptions [e.g., 18,23,24]. Extant research has reflected on a number of potential reasons that explain the persistence of this gender gap, including productivity, citation practices, co-authorship, network dynamics, publication venues, and differences in topics or methodologies.

First, research productivity stands out as one of the most prominent factors. The link between research output and impact is well-documented [5,21], and women have consistently been found to publish significantly less than men both at the field level and among top producers [2,16,25]. Van den Besselaar and Sandström [15] argue that women are caught in a vicious cycle that makes the academic glass ceiling hard to break: female researchers tend to have lower academic positions and are less often in a leading role. This negatively affects their performance, as those in higher positions tend to be more productive, which ultimately reinforces their lower status and position. Several studies have suggested that the productivity gap begins early in academic careers when researchers start a family and have children. This affects women’s careers more than men’s, as women continue to bear a majority of domestic responsibilities and child-care duties [5] and are more likely to experience higher levels of work-family conflicts [26,27].

Another contributing factor is differences in citation practices [3]. For instance, King et al. [28] revealed that male researchers are more likely to self-cite than women and that women are more likely than men to not cite their own previous work at all across fields. This tendency may increase men’s visibility and overall impact, as some evidence suggests that self-citations significantly increase future citations from others [29,30]. Additionally, research has highlighted gender homophily in citations, that is, the tendency of male and female researchers to cite publications of their own gender [31–33]. Given the higher proportion of men in most disciplines, this trend acts to the detriment of women, although it is difficult to discern whether this reflects an actual bias against the work of female researchers [33,34].

Regarding collaborations, studies show that while male researchers tend to work in larger and more international research networks that receive more funding [2,35], women participate in smaller networks and occupy authorship positions associated with seniority less frequently [7,10,11]. These trends disadvantage women, as larger and more internationally diverse teams tend to have higher research impact [36]. Relatedly, research indicates that men are more likely to publish in prestigious journals with a high impact factor [6,7]. For instance, a study among German full professors in Psychology found that men publish more often in top and standard journals than women [22]. Another study of articles from 54 journals listed in the Nature Index showed that 39% of female authors are responsible for 29.8% of all authorships [6].

Finally, men and women generally pursue different research topics and employ different methodologies [3,34,37]. While women tend to favor exploratory and qualitative methods, men are more inclined to use quantitative methodologies, which are more frequently published in high-impact journals [3,38]. Moreover, studies have shown that gender stereotypes persist in certain research areas, leading to significant inequalities in citation patterns and perceptions of quality in gender-typed research areas [34,39].

## The gender gap among highly cited researchers

As a result of the aforementioned disparities and barriers, the underrepresentation of women among the most frequently cited scholars becomes a significant consequence [14,17,40]. In science, productivity, research impact, and reputation are highly skewed: a small influential number of scholars account for a large share of publications and attract the majority of citations [2,14,17]. Therefore, top researchers play a crucial role. They publish significantly more than other individuals in similar positions and are highly influential to other researchers, often serving as role models and mentors [16]. They are also at the forefront of new methodological and theoretical developments in disciplines [34] and are the ones publishing the majority of highly cited publications [15]. Thus, examining the gender gap in this selective group of scholars is relevant because it can offer insightful information on the development of science and the overall state of knowledge production [14,34].

In this study, we extend previous research on gender differences in scholarly impact by examining the existing gender gap among highly cited researchers. We do not focus on identifying the underlying factors of this gap (summarized in the previous section), but on providing a comprehensive picture of the gender differences among this group of researchers across fields. So far, previous studies have shown that the representation of top female researchers is “worryingly low” compared to their proportion in the general population of researchers and senior scholars [14] and that it decreases as more elite ranges of performance are considered (top 10%, 5%, and 1%) [16]. Additionally, this underrepresentation varies strongly across countries and fields. Chan and Torgler [17] note that Finland has the highest proportion of top female researchers (20.45%) while Saudi Arabia has the lowest (2.08%). They also find that the lowest share of top women scientists is in Mathematics and Statistics, Engineering, and Physics and Astronomy.

Overall, these studies have provided extensive evidence on the gender gap among highly cited researchers. However, they have either focused on specific countries [18,40,41], examined a limited number of fields [16,17,42], or have not controlled for variables such as productivity [13,14]. Conversely, our study aims to examine gender differences in impact and citations among the most cited scholars in all fields, distinguishing between continents and controlling for research productivity and research experience (i.e., career length). It is important to control for these variables because they are strong predictors of research impact. Productivity is a “determining factor for being among the highly cited researchers” [40, p. 5805]. Likewise, various studies have suggested that the gender gap in productivity and impact can largely be explained by differences in research experience [2,15,18]. Male researchers tend to have longer careers and occupy higher positions, and those in higher positions are more productive. Thus, based on this literature review, we ask the following research questions:

RQ1: Assuming equal gender proportions (50%) among the most cited researchers, are there statistically significant differences in the a) pooled sample, b) continent-level, and c) field-level gender distribution?

RQ2: Are there statistically significant gender differences in a) citations and b) h-index in the ab_1_) pooled sample, ab_2_) continent-level and ab_3_) field-level?

RQ3: After controlling for research productivity and research experience, are there statistically significant gender differences in a) citations, and b) h-index at ab_1_) the pooled sample, ab_2_) continent-level, and ab_3_) field-level?

## Measurements

To answer the research questions, we collected the following measurements of the most cited authors of all scientific fields: name, scientific field, number of citations, research productivity, research experience, research impact, continent, and gender. Scientific fields were obtained from JCR categories which were then used to search the top authors on Google Scholar. Thus, we obtained authors’ name, citations, research productivity, research experience and research impact from Google Scholar. Research productivity was the number of publications listed in Google Scholar. Research experience was computed as the number of years from the first citation. Research impact was the h-index score reported by Google Scholar. In this study, citation counts were obtained as full counts from Google Scholar’s “Cited by” metrics, reflecting the total scholarly impact reported by the platform without fractionalization. We did not apply field-normalization to these citation metrics due to the challenges of standardizing across Google Scholar’s broad and heterogeneous coverage, which includes non-peer-reviewed materials such as theses, books, and gray literature, and varies significantly across disciplines (e.g., economics vs. sociology). This approach ensures consistency with Google Scholar’s raw data but may influence cross-disciplinary comparisons. Continent was inferred from the institutional email included in the authors’ Google Scholar profile. Gender was inferred using the GenderAPI online service.

Since its foundation in 2004, Google Scholar has gained worldwide popularity as a source for scientific information, becoming a central part of academic practice [43–45]. This search engine has numerous strengths: it is free, easy to use, can rapidly incorporate new academic information, and offers a wide range of coverage [44,46]. Additionally, it is the first tool most scientists use to carry out literature searches [47], and a common resource for quickly estimating researchers’ quality and impact [48].

However, it is important to acknowledge that numerous scholars have raised concerns about the reliability and reproducibility of bibliometric data derived from it [49–52]. One of the main criticisms is that its broad coverage stems from its indexing of all academic output, regardless of peer review. This includes non-journal work such as theses and dissertations, gray literature, books, conference proceedings, and unpublished materials, which inflate citation count and h-indexes [43,46,53], and make the data more susceptible to errors [43,52]. It has also been demonstrated that Google Scholar has duplicated records and that dishonest authors can easily manipulate their bibliometric indicators [51,53,54]. Moreover, Google Scholar lacks transparency and may yield results that are not always consistent or easily reproducible [49,50,53].

Despite these limitations, many of which stem from its automated approach to indexing [55], we decided to use Google Scholar as the database for this study for various reasons. First, previous research has shown that it is reliable in terms of coverage and citations, especially in fields such as social sciences, arts and humanities, and engineering and computer science, where Web of Science and Scopus do not provide exhaustive coverage [52,53]. For instance, strong correlations have been found between the citation counts of Google Scholar and those of Web of Science and Scopus [e.g., 47,55,56]. Second, as emphasized, it is one of the most widely used search engines among researchers and professionals [44,45], and it offers a broader and more international coverage due to its inclusion of non-English, free, and open-access work [53,57]. Finally, research suggests that Google Scholar is being used to assess the quality of scholars’ work and to inform decisions related to promotion, tenure, or grant applications [44,46,52,58].

## Data collection and processing

The data collection process for this study, conducted from September 26–28, 2023, involved automated data collection of publicly available Google Scholar profiles. To ensure compliance with Google Scholar’s terms of service, the data gathering process was designed to be non-disruptive, incorporating a timer between requests to limit the frequency of queries and minimize server load. All data extracted, including author names, affiliations, email domains, research labels, citation metrics, and publication counts, were obtained from publicly accessible profiles in accordance with ethical research practices and Google Scholar’s guidelines for non-commercial academic use. The data collection and processing included the following stages: (1) translating JCR categories into Google Scholar search terms, (2) running searches and collecting data from Google Scholar, (3) validation of categories, and (4) inferring additional measurements.

The JCR classifies journals in 254 categories. Since authors in Google Scholar declare labels of their scientific fields that differ from JCR categories, these were initially translated into Google Scholar search terms using the following rules: Categories representing a single scientific field (*N* = 108) did not require any change. Categories including two text strings separated with a comma (*N* = 65) were translated into Googler Scholar search terms by including the second term before the first when they represented a name and an adjective respectively (e.g., ‘Physics, Applied’ into ‘Applied Physics’), or by removing the first term when it represented a wider field of activity (e.g., ‘Computer Science, Artificial Intelligence’ into ‘Artificial Intelligence’). Remaining categories (*N* = 81) grouped multiple scientific fields separated by commas or ampersands, which were translated into search terms using ‘OR’ to separate the fields (e.g., ‘Anatomy & Morphology’ into ‘Anatomy OR Morphology’) while taking care of possible adjectives qualifying more than one field (‘Nuclear Science & Technology’ into ‘Nuclear Science OR Nuclear Technology’). The Supporting Information 1 S1 File presents all original JCR categories and their corresponding Google Scholar search terms.

As a second stage, we ran a Google Scholar search for each translated category (scientific field) to get the profile of the top authors. Profiles in Google Scholar include three fields which are the author’s name, affiliation, and labels. Labels are the author’s self-reported research fields and specialties. We used the ‘all-fields’ search which returns profiles where any of the profile fields matches the search term, including minor variations. The alternative ‘labels-only’ returns only exact matches of the labels text field, leaving out relevant authors. Initially we gathered the top 125 authors for each category. Since later the validation stage removes duplicated cross-listed authors, we wanted to have at least 100 authors for each category. Top authors and their measurements (citations, research productivity and research experience) were automatically collected with a Python script using the Selenium Web Driver with the web browser integrated to navigate and interact with the website programmatically.

In the third stage we validated, tuned, and excluded categories after individually checking collected data. Firstly, most authors do not report multidisciplinary and interdisciplinary categories, so they did not return enough results. Hence the terms ‘Multidisciplinary’ (*N* = 8) and ‘Interdisciplinary’ (*N* = 1) were then removed from these categories. Secondly, we removed specific terms from the following three categories because they returned results from unrelated fields: for ‘Computer Science, Hardware & Architecture’, we removed ‘Architecture’; for ‘Materials Science, Characterization & Testing’, we removed ‘Testing’; and for ‘Materials Science, Coatings & Films’, we removed ‘Films’. After tuning categories’ names, we found that eight were overlapping with other categories, and they were excluded. Fifty-two categories did not return at least 125 authors and were also excluded. Since this study focuses on highly cited authors, if a search term corresponding to a research field does not return a significant number of authors in Google Scholar, we considered that it is not well defined or representative of the top authors in that field. The Supporting Information 1 S1 File presents all original JCR categories, their corresponding Google Scholar search terms, adjustments made and exclusion criteria.

In the final stage, we inferred additional data (continent and gender) of authors and performed final checks. Data collection resulted in an initial dataset of 23,028 authors. Firstly, we noticed that 1,163 authors (5.05%) were duplicated, i.e., cross-listed in more than one category, and they were removed. Continent was determined using institutional emails as reported in Google Scholar and checking the university-domains-list GitHub repository (https://github.com/Hipo/university-domains-list) to determine the country. If the university domain was not found in this list, then the first level of the email domain was used comparing it to the list of country code top-level domains. Continent was then inferred from country. The affiliation country of 1,069 authors (4.97%) could not be determined and was coded as missing. Gender was inferred with the Gender-API web service using the feature that includes localization (country), which improves accuracy. The gender of 356 authors (1.55%) could not be determined and they were removed.

The final dataset included 21,509 authors from 194 scientific fields. Fields were also grouped at the field-level using the JCR scientific areas resulting in the following distribution: Arts & Humanities (6.84%), Engineering (12.82%), Exact sciences and physics (13.32%), Medicine (21.03%), Natural Sciences (25.92%) and Social Sciences (20.06%). The Supporting Information 2 S2 File presents the final preprocessed dataset used in this study from data extracted from Google Scholar public profiles. The dataset only includes processed, anonymized, or aggregated data derived from public profiles, not direct reproductions of Google Scholar’s interface or proprietary content.

The data collection and preprocessing pipeline for gathering and refining data from Google Scholar profiles of highly cited researchers is detailed in the S3 File Online Appendix. This appendix outlines the methodology used to translate JCR categories into search terms, extract author profile data, and preprocess the dataset through steps such as deduplication, name and affiliation standardization, gender inference, and geographic classification. The pipeline addressed data quality concerns raised by Martín-Martín et al. [57] by removing duplicates, handling missing citations, standardizing affiliations via email domains and ROR, and ensuring temporal accuracy. Limitations of Google Scholar, such as the inclusion of non-peer-reviewed content and unverified profiles, were mitigated through validation but not fully eliminated, as discussed later in the limitations.

## Analysis strategy

Various statistical techniques were employed to address the research questions. To specifically address RQ1, a chi-square goodness-of-fit test was conducted, assuming equal gender proportions (i.e., 50% men and 50% women), for a) general (all authors in the sample), b) continent, and c) field-level analyses. In addition, since h-index and citations scores were not normally distributed, to answer RQ2 the rank-based nonparametric test Mann-Whitney U test was implemented. Finally, to address RQ3 a series of bootstrapped hierarchical ordinary less-square regression were implemented.

When addressing RQ3 and aiming to robustly isolate the potential association of gender with both citation scores and h-index, we opted to control for research productivity and research experience, as prior literature has consistently shown that higher productivity is associated with higher citation scores and potentially higher h-index values [12,40] and considering that years of experience may also be associated with levels of impact in scientific fields [2,15]. Accordingly, our models controlled for both. In addition, in order to account for possible overlap between citations and h-index in assessing research impact, we controlled for the effect of each when the other was examined. In addition, as robustness-check, we ran a series of bootstrapped hierarchical OLS regression, removing h-index when predicting citations and citations when predicting h-index. Results can be found in the S3 File Online Appendix (Tables A1 to A4).

In the main analysis and the robustness-checks, the series of bootstrapped hierarchical ordinary less-square regression accounted for robust standard errors based on bootstrapping to 1000 resamples with biased corrected confidence intervals set at 95% to assess statistical significance. We employed bootstrapping as a statistical technique because the dependent variables exhibited non-normal distributions. Bootstrapping, a resampling technique, operates without reliance on assumptions about data distribution [59]. It involves generating multiple samples with replacement from the original dataset, allowing for regression analyses on each resampled dataset. This method is particularly advantageous when dealing with non-normally distributed data or when concerns arise about the assumptions of traditional statistical tests [60]. Creating multiple resampled datasets enables the derivation of robust estimates for standard errors, confidence intervals, and p-values, even in cases where the underlying distribution deviates from normality.

## Results

RQ1 inquired if assuming equal gender proportions (50%) among the most cited authors in Google Scholar, there were statistically significant differences in the a) pooled sample, b) continent-level, and c) field-level gender distribution. Results of RQ1a reported in Table 1, revealed that 0 cells have expected frequencies less than 5. In this case, the minimum expected cell frequency is 10754.6. The chi-square goodness-of-fit test indicated that the gender distribution in the pooled sample were not equally represented (χ^2^(1) = 7559.06, *p* = .000), as there were 6375.5 females fewer than expected (N_males_ = 17130; N_females_ = 4379). Accordingly, in the pooled sample, there are statistically significant fewer female researchers than male researchers, assuming an equal gender distribution. Specifically, the ratio is 0.255 women for 1 man.

**Table 1:** Gender distribution among the most cited scholars in Google Scholar at different levels of analysis.

Level of Analysis	Male	Female	Expected	Residual	Total	Chi-square
Pooled sample	17130	4379	10754.5	−6375.5	21509	χ^2^(1) = 7559.06, *p* = .000
*Continent-level*						
Africa	329	72	200.5	−128.5	401	χ^2^(1) = 164.71, *p* = .000
Asia	3245	560	1902.5	−1342.5	3805	χ^2^(1) = 1894.67, *p* = .000
Europe	6268	1652	3960	−2308	7920	χ^2^(1) = 2690.33, *p* = .000
North America	5588	1652	3620	−1968	7240	χ^2^(1) = 2139.79, *p* = .000
South America	176	60	118	−58	236	χ^2^(1) = 57.01, *p* = .000
Oceania	631	207	419	−212	838	χ^2^(1) = 214.53, *p* = .000
*Field-level*						
Arts & Humanities	1032	439	735.5	−296.5	1471	χ^2^(1) = 239.05, *p* = .000
Social sciences	3188	1127	2157.5	−1030.5	4315	χ^2^(1) = 984.40, *p* = .000
Natural sciences	4546	1030	2788	−1758	5576	χ^2^(1) = 2217.04, *p* = .000
Medicine	3532	992	2262	−1270	4524	χ^2^(1) = 1426.08, *p* = .000
Engineering	2372	386	1379	−993	2758	χ^2^(1) = 1430.09, *p* = .000
Exact sciences & Physics	2460	405	1432.5	−1027.5	2865	χ^2^(1) = 1474.00, *p* = .000

*Note.* Residuals are computed for female scholars.

Similar patterns are observed in the gender distribution at the continent level. The findings indicate that across all continents (Africa: χ^2^(1) = 164.71, *p* = .000, Asia: χ^2^(1) = 1894.67, *p* = .000, Europe: χ^2^(1) = 2690.33, *p* = .000, North America: χ^2^(1) = 2139.79, *p* = .000, South America: χ^2^(1) = 57.01, *p* = .000, and Oceania: χ^2^(1) = 214.53, *p* = .000), under the assumption of equal gender distribution, there is a statistically significant underrepresentation of women among the most highly cited scholars in Google Scholar. As indicated in Table 1, the underrepresentation of female scholars is pervasive, with particularly noteworthy disparities observed in Asia (0.172 females per male) and Africa (0.218 females per male).

Parallel to both the pooled sample and continent-level analysis, the scrutiny of field-level differences unveiled that, under the assumption of equal gender distribution among the most cited scholars, female scholars are statistically significantly less represented than their male counterparts across all scientific fields (Arts & Humanities: χ^2^(1) = 239.05, *p* = .000, Social sciences: χ^2^(1) = 984.40, *p* = .000, Natural sciences: χ^2^(1) = 2217.04, *p* = .000, Medicine: χ^2^(1) = 1426.08, *p* = .000, Engineering: χ^2^(1) = 1430.09, *p* = .000, and Exact sciences & Physics: χ^2^(1) = 1474.00, *p* = .000). The most prominent gender dis*p*arities in terms of ratios are observed in engineering (with a ratio of 0.162 women per man) and in exact sciences & physics (with a ratio of 0.164 women per man). The comparatively more balanced gender distribution, albeit still far from achieving gender equality, is found in Arts & Humanities, where the women ratio is 0.425 per man.

RQ2 investigated the presence of statistically significant gender differences in both citations and h-index across various levels of analysis. As depicted in Table 2, with the exception of South America, statistically significant gender differences in citations were observed in the pooled sample and across all other continents and fields. In each of these instances, men exhibited significantly higher citation counts scores compared to their female counterparts. Similarly, the analyses showed that female researchers have a significantly lower h-index than male researchers in all fields and geographic regions, except in South America and Africa.

**Table 2:** Gender differences in citations and H-index among the most cited scholars in Google Scholar at different levels of analysis.

Level of Analysis	Citations			H-index		
	U	Z	p	U	Z	p
Pooled sample	30724508	−18.49	.000	29640915	−21.45	.000
*Continent-level*						
Africa	9790	−2.30	.021	10275	−1.76	.078
Asia	751363.50	−6.55	.000	722294	−7.76	.000
Europe	3960722	−14.71	.000	3833913.50	−16.25	.000
North America	3651788	−12.91	.000	3546853.50	−14.32	.000
South America	4723.50	−1.21	.223	4729	−1.20	.227
Oceania	49968.50	−5.07	.000	46253.50	−6.30	.000
*Field-level*						
Arts & Humanities	141549.50	−11.39	.000	137356.50	−11.96	.000
Social sciences	1545979	−6.96	.000	1523945	−7.58	.000
Natural sciences	1958741	−8.19	.000	1873406	−10.02	.000
Medicine	1423070.50	−9.04	.000	1390709.50	−9.93	.000
Engineering	366137	−6.31	.000	360935.50	−6.67	.000
Exact sciences & Physics	408323.50	−5.82	.000	392506	−6.84	.000

Nevertheless, when scrutinizing gender disparities in citations and h-index across various levels of analysis while accounting for factors such as research productivity, research experience, and h-index (RQ3), the scenario unfolds differently. First, concerning a_1_) citations in the pooled sample, the regression analysis revealed that female scholars are statistically significant more cited than male (*b* = 1538.81, *p* < .001). At a_2_) continent level, female researchers are statistically more cited than men in Asia (*b* = 1869.87, *p* < .01), and Europe (*b* = 1499.22, *p* < .01). In Africa, North America, South America, and Oceania, citation scores between women and men were not statistically significant, as reported in Table 3.

**Table 3:** Bootstrapped OLS regression testing the association between author gender and citation scores in the pooled sample and continent-level.

	Pooled sample	Continent-level, *b* (SE)					
*Block 1: Scientometric controls*		Africa	Asia	Europe	North America	South America	Oceania
Research productivity	−0.50 (1.20)	−13.84 (7.24)	4.27*** (1.04)	−2.29*** (2.99)	0.85 (2.21)	−8.21 (5.02)	−16.12 (10.91)
Research experience	−402.54*** (30.20)	−455.91 (167.67)	−342.51*** (38.78)	−525.19*** (62.92)	−409.35*** (2.21)	−483.28* (155.27)	−537.35*** (101.72)
H-index	805.46*** (26.35)	1064.69* (344.39)	598.39*** (572.71)	826.54*** (72.25)	820.02*** (22.70)	678.62* (143.80)	931.76*** (172.73)
*ΔR*^*2*^	56.5%	57%	65.1%	52.8%	58.6%	49.3%	53.2%
Block 2: Variable of interest							
Gender_(female)_	1548.81*** (272.61)	−1064.69 (344.39)	1869.87** (572.71)	1499.22** (441.06)	963.78 (503.61)	−1444.35 (1035.40)	4172.44 (2036.98)
ΔR^2^	0%	0%	0.1%	0%	0%	0.1%	0.3%
Total R^2^	56.5%	57%	65.2%	52.8	58.6%	49.3%	53.5%
Adjs. R^2^	56.5%	56.6%	65.2%	52.8%	58.6%	49.4%	53.3%
Residual Std. Error	21562.60	19738.30	12267.66	22564.89	22748.83	120550.38	22127.88
N	21509	401	3805	7920	7240	236	838

*Note*. Cell entries of citations are final-entry unstandardized beta (*b*) coefficients. Coefficients effects accounted for robust standard errors based on bootstrapping to 1,000 resamples with biased corrected confidence set at 95% to assess statistical significance. Bootstrapped standard errors in brackets. * *p* < .05, ** *p* < .01, *** *p* < .001

Regarding a_3_) citations at field level, the regression analysis unveiled statistically significant gender differences in natural sciences (*b* = 1815.23, *p* < .001), as well as in exact sciences and physics (*b* = 2303, *p* < .01), but not in the remaining fields, as reported in Table 4.

**Table 4:** Bootstrapped OLS regression testing the association between author gender and citation scores at field-level.

	Field-level, *b* (SE)					
*Block 1: Scientometric controls*	Arts & Humanities	Social sciences	Natural sciences	Medicine	Engineering	Exact sciences & Physics
Research productivity	3.16 (5.86)	1.13 (4.86)	1.02 (1.16)	−1.83 (1.33)	2.22* (0.93)	2.43 (2.97)
Research experience	−290.01 (231.11)	−519.06*** (85.01)	−234.96*** (1.16)	−725.37*** (55.55)	−68.19* (28.74)	−551.27*** (65.02)
H-index	993.68 (337.75)	1034.86*** (80.02)	668.11*** (31.48)	828.44*** (28.69)	499.24*** (16.53)	908.20*** (53.20)
*ΔR* ^*2*^	36.8%	52.9%	63.1%	70.1%	73.1%	63.8%
Block 2: Variable of interest						
Gender_(female)_	6041.30 (3245.11)	−823.51 (702.09)	1815.23*** (389.87)	535.61 (557.11)	732.91 (399.05)	2303.31** (827.59)
ΔR^2^	0.4%	0%	0.1%	0%	0%	0%
Total R^2^	37.2%	52.9%	63.2%	70.1%	73.1%	63.9%
Adjs. R^2^	37%	52.9%	63.1%	70.1%	73.1%	63.8%
Residual Std. Error	31400.11	28631.17	13653.76	17185.84	8839.94	24136.32
N	1471	4315	5576	4524	2758	2865

*Note*. Cell entries of citations are final-entry unstandardized beta (*b*) coefficients. Coefficients effects accounted for robust standard errors based on bootstrapping to 1,000 resamples with biased corrected confidence set at 95% to assess statistical significance. Bootstrapped standard errors in brackets. * *p* < .05, ** *p* < .01, *** *p* < .001

Once again, the scenario differs when scrutinizing the h-index, accounting for research productivity, experience, and citation scores. Regarding gender differences in b_1_) h-index in the pooled sample, the regression analysis revealed that female scholars have a statistically significant lower h-index compared to their male counterparts (*b* = −2.01, *p* < .001). Testing gender differences b_2_) in h-index at continent-level, the regression analysis revealed statistically significant gender differences in Asia (*b* = −3.18, *p* < .001), Europe (*b* = −1.84, *p* < .001), North America (*b* = −2.24, *p* < .001), and Oceania (*b* = −4.76, *p* < .001), but not in Africa and South America. In the countries in which gender differences in h-index were statistically significant, in all cases, female scholars have less h-index than males (Table 5).

**Table 5:** Bootstrapped OLS regression testing the association between author gender and h-index in the pooled sample and continent-level.

	Pooled sample	Continent-level, *b* (SE)					
*Block 1: Scientometric controls*		Africa	Asia	Europe	North America	South America	Oceania
Research productivity	0.03*** (0.00)	0.03*** (0.00)	0.01*** (0.00)	0.04*** (0.00)	0.04*** (0.00)	0.03*** (0.00)	0.04*** (0.00)
Research experience	0.65*** (0.01)	0.30*** (0.07)	0.45*** (0.03)	0.61*** (0.02)	0.58*** (0.02)	0.50*** (0.10)	0.37*** (0.08)
Citations	0.00*** (4.36E-005)	0.00*** (.000)	0.00*** (4.62 E-005)	0.00*** (9.31 E-005)	0.00*** (3.69 E-005)	0.00** (0.00)	0.00*** (8.89E-005)
*ΔR* ^*2*^	71.2%	71.7%	70.2%	72.5%	74.2%	72.6%	72.6%
Block 2: Variable of interest							
Gender_(female)_	−2.01*** (0.25)	3.49 (1.63)	−3.18*** (0.62)	−1.84*** (0.39)	−2.24*** (0.45)	−0.73 (1.50)	−4.76*** (1.18)
ΔR^2^	0.1%	0.3%	0.2%	0.1%	0.1%	0%	0.4%
Total R^2^	71.3%	72%	70.4%	72.5%	74.3%	72.6%	73%
Adjs. R^2^	71.3%	71.7%	70.3%	72.5%	74.3%	72.2%	72.9%
Residual Std. Error	17.24	12.74	14.87	16.34	17.27	10.95	15.53
N	21509	401	3805	7920	7240	236	838

*Note*. Cell entries of h-index are final-entry unstandardized beta (*b*) coefficients. Coefficients effects accounted for robust standard errors based on bootstrapping to 1,000 resamples with biased corrected confidence set at 95% to assess statistical significance. Bootstrapped standard errors in brackets. * *p* < .05, ** *p* < .01, *** *p* < .001

Finally, examining gender differences b_3_) in h-index at field level, the regression analysis revealed statistically significant gender differences in all scientific field, except for social sciences: in arts and humanities (*b* = − 5.28, *p* < .001), natural sciences (*b* = −2.48, *p* < .001), medicine (*b* = −0.99, *p* < .05), engineering (*b* = −1.61, *p* < .05), and exact sciences and physics (*b* = −2.48, *p* < .01). Once again, in all instances, female scholars exhibit a statistically significant lower h-index than their male counterparts, as reported in Table 6.

**Table 6:** Bootstrapped OLS regression testing the association between author gender and h-index at field-level.

	Field-level, *b* (SE)					
*Block 1: Scientometric controls*	Arts & Humanities	Social sciences	Natural sciences	Medicine	Engineering	Exact sciences & Physics
Research productivity	0.03*** (0.00)	0.03*** (0.00)	0.01*** (0.00)	0.02*** (0.00)	0.02*** (0.00)	0.03 (0.00)
Research experience	0.45*** (0.07)	0.67*** (0.03)	0.59*** (0.03)	0.95*** (0.03)	0.38*** (0.03)	0.51 (0.03)
Citations	0.00** (0.00)	0.00*** (4.00E-005)	0.00*** (7.57E-005)	0.00*** (3.05E-005)	0.38*** (0.03)	0.00*** (6.44E-005)
*ΔR* ^*2*^	60%	69.8%	71.2%	80.7%	78.9%	76.6%
Block 2: Variable of interest						
Gender_(female)_	−5.28*** (0.90)	−0.39 (0.51)	−2.48*** (0.47)	−0.99* (0.51)	−1.61* (0.63)	−2.48** (0.84)
ΔR^2^	0.9%	0%	0.1%	0%	0%	0.1%
Total R^2^	60.9%	69.8%	71.3%	80.7%	78.9%	76.7%
Adjs. R^2^	60.8%	69.8%	71.3%	80.7%	78.9%	76.7%
Residual Std. Error	15.69	16.99	14.83	15.43	13.19	18.02
N	1471	4315	5576	4524	2758	2865

*Note*. Cell entries of h-index are final-entry unstandardized beta (*b*) coefficients. Coefficients effects accounted for robust standard errors based on bootstrapping to 1,000 resamples with biased corrected confidence set at 95% to assess statistical significance. Bootstrapped standard errors in brackets. * *p* < .05, ** *p* < .01, *** *p* < .001.

## Discussion and conclusions

The findings of this study draw on a unique dataset of 21,509 authors from Google Scholar, highlighting the differences in representation, citations, and impact among highly cited researchers across all disciplines. Overall, the study shows that women are significantly underrepresented among top researchers and exhibit significantly lower citation rates and h-index compared to men. When accounting for research productivity and experience, the findings reveal that women are cited significantly more than their male counterparts. However, female researchers show a significantly lower h-index. This suggests a higher volume of citations for female scholars, but a lower long-term impact as measured by the h-index.

First, the underrepresentation of women among the highly cited researchers of all disciplines (RQ1a; 0.255 women per man in the pooled sample) is in line with studies in the area [e.g., 13,14], and evidences that gender parity in scholarly representation remains a distant goal. Regarding geographical regions (RQ1b), the share of women in Asia (0.172 per man) and Africa (0.218 per man) is remarkably low. This may be attributed to the challenges that women encounter in accessing the education system and academia in low-income African countries [61] and the gender imbalances that persist in Asian societies, which are more pronounced than in other continents like Europe or North America [62]. Moreover, the disciplines with the lowest representation of female top researchers are in STEM fields: engineering, with a ratio of 0.164 women per man, and exact sciences and physics, with a ratio of 0.165 women per man (RQ1c). This finding is consistent with extensive evidence highlighting the low presence of women in STEM disciplines [16,37,63].

The underrepresentation of women in academia, both generally and among top researchers specifically, are systemic and influenced by several factors [64]. A prominent one is the gender productivity gap, which emerges early in academic careers and tends to persist over time [5,18]. Notably, gender represents the most discussed driver of the cumulative advantage in science [65,66], a process through which early success leads to increasing recognition and benefits. Women often begin their careers at a disadvantage, facing obstacles such as imbalances in the division of domestic responsibilities [27], which may result in less time for research activities and thus lower productivity [5]. Other important barriers are demographic inertia [14,67], smaller and less international collaboration networks [2,35], discrimination towards gendered topics [34], or the well-known Matilda effect [68]. Other studies have pointed out that senior academic positions typically generate the highest levels of productivity, impact, and citations [12,15]. This disparity disadvantages women, who often face delays in attaining these positions or may not achieve them at all. In STEM fields in particular, a hostile or “chilly” culture towards women often prevails, discouraging them from becoming professors and increasing their intentions to leave academia [63,69].

The findings relating to RQ2 show that women receive fewer citations and have less impact at the aggregate level, except in terms of citations in South America and the h-index in South America and Africa, where the relationships were not statistically significant. However, the results of RQ3 show that, when taking into consideration productivity and career length—two crucial variables for being among the highly cited authors [5,40]—women are significantly more cited (RQ3_a1_). These results are in line with other studies [e.g., 6,17], and imply that women receive citations *per article* at a comparable or even higher rate than men [18,70]. These results also suggest that increasing the number of female researchers could help to improve their number among highly cited researchers [17], which emphasizes the need to create initiatives that support women in academia and that counteract the systemic barriers that they deal with.

Specifically, the findings show that women receive significantly more citations in Asia and Europe (RQ3_a2_) and in the disciplines of natural sciences and exact sciences and physics (RQ3_a3_). This may be due to a range of factors. The gender advantage observed in Europe could be a result of the policies implemented by the European Union and some European countries over the past decade to achieve gender balance [17,71]. In contrast, the findings from Asia and the two STEM fields are unexpected, given that these contexts have some of the lowest proportions of female researchers. In this sense, it is important to acknowledge that these highly cited women probably constitute a select “survivor” group and might not reflect the experiences of the majority of women in those contexts [72].

Finally, our findings show that female researchers have a lower h-index across all regions except Africa and South America, and across all scientific fields except social sciences, even after controlling for productivity and career length (RQ3b). This is likely due to the fact that women generally have a smaller overall scholarly output. As a result, although they may have a high number of citations, these citations are likely concentrated in fewer papers, which ultimately yields a lower h-index. This could also be a consequence of shorter academic careers. Thus, the use of the h-index for hiring and promotion decisions is problematic, as it may partially reflect the systemic gender differences and structural barriers present in academia [73], that make it more challenging for women to publish more papers [74].

Taken together, our findings advance previous research by providing a comprehensive, multidisciplinary view of the gender gap among highly cited researchers. Previous research has often focused on specific fields [16,41] or individual countries [18,40]. Even the broadest analyses have been limited to a relatively small number of fields (e.g., 21 fields in [17]) or focused only on representation rather than academic impact [13,14]. In contrast, this study provides a more extended perspective drawing on Google Scholar, a highly popular yet understudied search engine. In doing so, it emphasizes the complex dynamics of gender disparities in academia and the need for further research and interventions to address these issues.

## Limitations

It is crucial to note that while these findings offer valuable insights, some limitations are noteworthy. First, the study’s reliance on data from Google Scholar comes along the limitations and potential errors associated to this search engine, which have been widely discussed [e.g., 49,52,53]. Unlike other scientific databases such as Web of Science or Scopus, Google Scholar has a less stringent indexing policy, including both peer-reviewed journals and gray literature. This implies that authors’ impact and citations may be inflated, potentially introducing bias to the findings, and limiting their validity and comparability across disciplines and regions. In addition, in Google Scholar, authors create and manage their own profiles, which excludes from our sample researchers who either lack a profile or list different categories or subfields. Moreover, although our focus is scholarly impact, citations in lower quality documents may imply other kinds of impact, such as educational or societal [55]. It has also been noted that Google Scholar lacks transparency, standardization, and may include unverified author identities [49,50,52,53].

A further limitation of this study is the absence of field-normalization for citation metrics, which may affect the comparability of scholarly impact across macro fields due to Google Scholar’s inclusion of diverse publication types and disciplinary variations in citation practices. This constraint arises from the platform’s broad and heterogeneous coverage, which complicates standardization efforts. Future studies could address this by applying normalization techniques using databases like Scopus or Web of Science, which offer more controlled indexing, to facilitate standardized comparisons across disciplines. This acknowledgment underscores the need for cautious interpretation of our findings and highlights avenues for improving the rigor of bibliometric analyses.

Taken together, it is challenging to assess how these limitations and potential errors have influenced our findings, as currently there is no automated computational technique for revising this kind of data. We do not claim that the findings provide an exact depiction of reality, but rather that they reflect the information available on Google Scholar. Thus, we can assume that errors, if present, may be randomly distributed across the sample, and that the findings still provide a reasonably accurate depiction of the phenomenon. However, as it is clear that employing other databases may yield slightly different results, we recommend that future research draws on other databases such as Scopus or Web of Science to validate and extend our findings. To further validate our findings, future research could compare our results with those derived from curated datasets, such as the Stanford dataset of highly cited researchers, which offers greater standardization and a larger sample size. Such a comparison could enhance confidence in the observed gender disparities by mitigating some of the limitations associated with Google Scholar’s less stringent indexing policies.

Second, our study did not control for self-citations, which have been shown to significantly boost future citations from others [29,30]. Finally, since we obtained the fields from categories listed in JCR, our study may potentially be missing categories that are not included in this ranking. Future studies should examine other databases to ascertain and expand our findings, taking into consideration the potential influence of self-citations.

Finally, the use of the GenderAPI service for inferring author gender, while practical for large-scale analysis, introduces potential misclassification biases, particularly for non-Western or gender-neutral names. The binary classification approach (male/female) may oversimplify gender identities and fail to account for cultural naming conventions, leading to inaccuracies for 356 authors (1.55%) whose gender could not be determined and were excluded from gender-based analyses. This exclusion and potential misclassification may impact the validity of gender disparity comparisons, especially in fields with diverse author populations. For instance, studies have shown that automated gender inference tools can exhibit lower accuracy for non-Western names due to limited training data diversity [75,76]. Future research could mitigate these issues by incorporating multi-category gender frameworks or leveraging manual validation for ambiguous cases, though such approaches may be resource-intensive for large datasets. These limitations highlight the need for cautious interpretation of our gender-based findings and suggest opportunities for refining gender inference methodologies in bibliometric research.

## Supporting information

S1 File Supporting information 1. Dataset with JCR categories, their corresponding Google Scholar search terms, adjustments made and exclusion criteria.
(XLSX)

S2 File Supporting information 2. Dataset used in this study from data extracted from Google Scholar public profiles.
(XLSX)

S3 File Online Appendix. Data collection and preprocessing pipeline of google scholar researcher profiles, and additional analyses and robustness checks.
(DOCX)
