# Supplementary material for: Gender gap in citations, h-index, and representation: Examining the highly cited authors across continents and disciplines in Google Scholar
Source: PLoS One. 2025 Nov 13;20(11):e0334690. doi: 10.1371/journal.pone.0334690 (PMC12614618; doi:10.1371/journal.pone.0334690)
Supplement: S3 File — Data collection and preprocessing pipeline of google scholar researcher profiles, and additional analyses and robustness checks. (DOCX) [file pone.0334690.s003.docx]

**Online Appendix for “Gender Gap in Citations, H-Index, and Representation: Examining the Highly Cited Authors Across Continents and Disciplines in Google Scholar”**

**Data Collection and Preprocessing Pipeline of Google Scholar Researcher Profiles**

This appendix outlines the methodology for collecting and preprocessing data from Google Scholar profiles of highly cited researchers across 194 scientific fields. Conducted from September 26 to 28, 2023, the pipeline generated an initial dataset of 23,028 author profiles, refined to a final dataset of 21,509 unique authors through systematic extraction, cleaning, validation, and enrichment processes.

**Data Collection – Translating JCR Categories**

The pipeline began by mapping 254 Journal Citation Reports (JCR) categories to Google Scholar search terms, as described in the "Data Collection and Processing" section of the manuscript. Single-field categories (e.g., "Physics, Applied" to "Applied Physics") were retained or adjusted, while multi-field categories (e.g., "Anatomy & Morphology" to "Anatomy OR Morphology") were adapted using logical operators to align with Google Scholar's search syntax. Supplementary Material 1 provides a complete list of translations.

**Data Collection –Extraction**

Data extraction utilized Selenium WebDriver to navigate Google Scholar’s dynamic webpages. Data gathering was conducted responsibly (e.g., with timers to avoid server overload) and for non-commercial academic purposes. For each scientific field, a search was executed using the URL:

[https://scholar.google.com/citations?view_op=search_authors&mauthors={search-term}](https://scholar.google.com/citations?view_op=search_authors&mauthors=%7bsearch-term%7d)

**
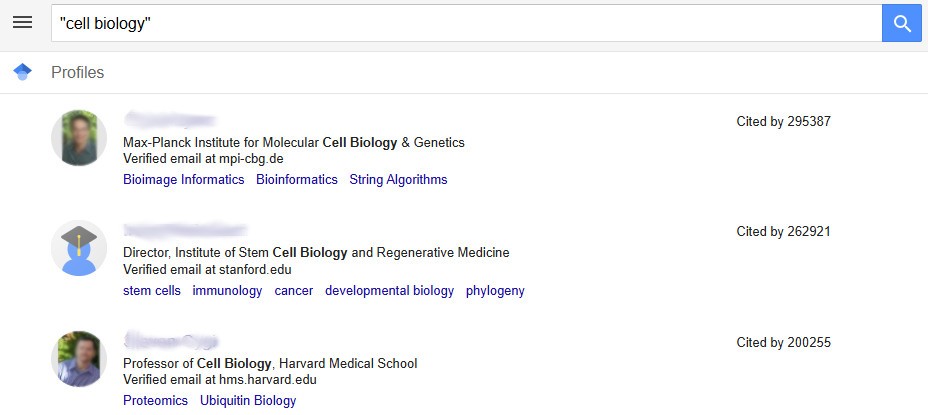
**Search terms were enclosed in double quotes, with multiple terms separated by "OR" when applicable. The resulting HTML was parsed to extract author profile links, iterating through paginated results by clicking the "Next" button until either 125 profiles per field were collected or no further results were available (see Figure 1).

*Figure 1: Google Scholar search results page for a scientific field. Author profiles were accessed sequentially, with the "Next" button clicked programmatically to retrieve up to 125 profiles per field.*

Note that each search term was included between double quotes and multiple search terms, when required, were separated by “OR”.

For each author profile, Selenium extracted data including name, affiliation, email domain, research labels, and citation metrics (total and last five years). The profile page was scrolled, and the "Show more" button was clicked iteratively to display the full publication list, which was parsed to determine the total number of publications (see Figure 2).

**
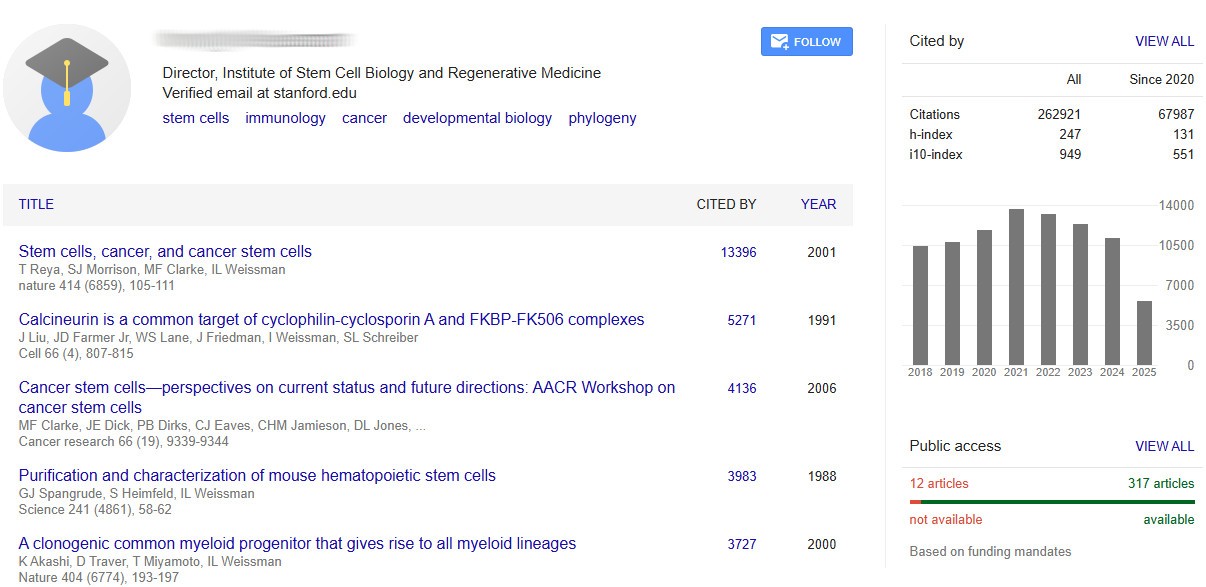
**

*Figure 2: Author profile page on Google Scholar. Name, affiliation, email domain, research labels, and citation metrics were extracted from the HTML. Publications were obtained by scrolling and clicking "Show more" to display the complete publication list.*

Citation history was accessed by clicking the "Cited by: VIEW ALL" button, with the history scrolled programmatically to extract the years of the first and last citations (see Figure 3). **
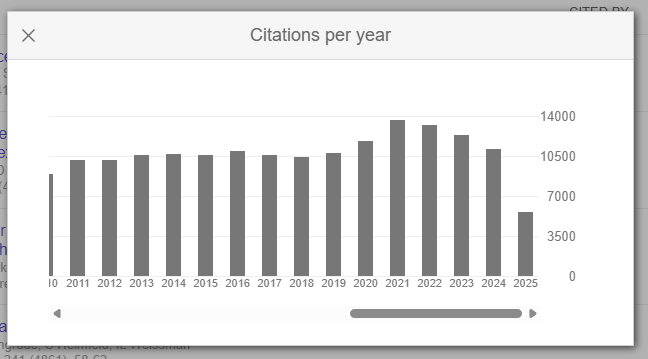
**

*Figure 3: Citation history accessed via the "Cited by: VIEW ALL" button on the author’s profile, scrolled programmatically to extract first and last citation years.*

**Preprocessing Pipeline**

The preprocessing pipeline refined the raw data through the following steps:

**1. Crosslisted Authors Identification**

Authors appearing in multiple fields were identified by detecting duplicate Google Scholar IDs. A total of 1,163 duplicates (5.05%) were flagged and removed to ensure a unique author dataset.

**2. Name Cleaning**

Author names were cleaned by removing titles (e.g., "Dr.", "Professor"), suffixes (e.g., "PhD"), alternative names following separators (e.g., "OR"), and excess whitespace. This standardization improved consistency for gender inference and addressed Google Scholar’s unstructured name data.

**3. Affiliation Standardization**

Affiliations were standardized using the Research Organization Registry (ROR) API (https://ror.readme.io/docs/rest-api). Unstructured affiliation strings were matched to ROR records, with hierarchical processing to identify root institutions, stopping at university systems (e.g., "California University System") or national government entities (e.g., "Government of the United States of America").

**4. Country Code Inference**

Country codes were derived from email domains using: (a) Country Code Top-Level Domains (ccTLDs), (b) the university domains list, or (c) ROR API country codes when domains were unmapped. The country for 1,069 authors (4.97%) could not be determined and was coded as missing.

**5. Gender Inference**

Gender was inferred using the Gender-API service with localization features to enhance accuracy. Authors with undetermined gender (356, 1.55%) were excluded to ensure data completeness for gender disparity analyses.

**6. Continent and Country Name Assignment**

Country codes were mapped to continents and country names using ISO 3166-1 alpha-2 standards, ensuring consistent geographic classifications for continent-level analyses.

**7. Field Grouping**

The 194 fields were grouped into 21 JCR-defined categories (e.g., Biology & Biochemistry) and further aggregated into six scientific areas: Arts & Humanities, Social Sciences, Natural Sciences, Medicine, Engineering, and Exact Sciences & Physics.

**Additional Analyses and Robustness Checks**

**Table A1.** *Bootstrapped* *OLS regression testing the association between author gender and citation scores in the pooled sample and continent-level without h-index*

|  | Pooled sample | Continent-level, *b* (SE) | | | | | |
| --- | --- | --- | --- | --- | --- | --- | --- |
| *Block 1: Scientometric controls* |  | Africa | Asia | Europe | North America | South America | Oceania |
| Research productivity | 45.49*** (1.78) | 50.90 (25.08) | 26.26*** (1.95) | 52.86*** (3.78) | 60.11*** (3.10) | 25.31* (6.21) | 47.96*** (5.30) |
| Research experience | 210.92*** (24.72) | -247.72 (156.08) | -151.23** (51.55) | -29.29 (41.66) | 108.36* (45.95) | -229.36 (151.55) | -335.11* (114.62) |
| *ΔR^2^* | 25.7% | 18.1% | 26.6% | 26.4% | 32.4% | 17.7% | 18.7% |
| Block 2: Variable of interest |  |  |  |  |  |  |  |
| Gender_(female)_ | -125.50 (360.48) | 4782.42 (2349.29) | -69.55 (724.10) | -35.99 (500.77) | -1430.78* (686.42) | -3136.77 (1491.80) | -459.14 (2366.70) |
| ΔR^2^ | 0% | 0.4% | 0% | 0% | 0% | 0.7% | 0% |
| Total R^2^ | 25.7% | 18.5% | 26.6% | 26.4% | 32.5% | 18.4% | 18.8% |
| Adjs. R^2^ | 25.7% | 17.9% | 26.5% | 26.4% | 32.4% | 17.3% | 18.5% |
| Residual Std. Error | 28191.21 | 27147.84 | 17822.88 | 28172.47 | 29068.93 | 15276.33 | 29233.18 |
| N | 21509 | 401 | 3805 | 7920 | 7240 | 236 | 838 |

*Note*. Cell entries of citations are final-entry unstandardized beta (*b*) coefficients. Coefficients effects accounted for robust standard errors based on bootstrapping to 1,000 resamples with biased corrected confidence set at 95% to assess statistical significance. Bootstrapped standard errors in brackets. * *p* < .05, ** *p* < .01, *** *p* < .001

**Table A2.** *Bootstrapped* *OLS regression testing the association between author gender and citation scores at field-level without h-index*

|  | Field-level, *b* (SE) | | | | | |
| --- | --- | --- | --- | --- | --- | --- |
| *Block 1: Scientometric controls* | Arts & Humanities | Social sciences | Natural sciences | Medicine | Engineering | Exact sciences & Physics |
| Research productivity | 53.38 (21.73) | 62.16*** (5.59) | 27.86*** (1.78) | 44.95*** (2.22) | 29.74*** (1.58) | -2.43 (2.91) |
| Research experience | 211.69 (97.26) | 290.13*** (71.06) | 348.24*** (32.71) | 140.22** (52.27) | 276.13*** (35.74) | -511.27*** (61.78) |
| *ΔR^2^* | 16.7% | 24.4% | 22.2% | 33% | 39.6% | 63.8% |
| Block 2: Variable of interest |  |  |  |  |  |  |
| Gender_(female)_ | 1044.06 (1976.85) | -1982.19* (932.14) | 328.57 (555.85) | -641.72 (839.16) | -165.89 (556.20) | 2303.32* (857.86) |
| ΔR^2^ | 0% | 0% | 0% | 0% | 0% | 0% |
| Total R^2^ | 16.7% | 24.4% | 22.2% | 33% | 39.6% | 63.9% |
| Adjs. R^2^ | 16.5% | 24.4% | 22.1% | 33% | 39.5% | 63.8% |
| Residual Std. Error | 36162.05 | 36284.91 | 19845.77 | 25718.65 | 13251.26 | 24136.32 |
| N | 1471 | 4315 | 5576 | 4524 | 2758 | 2865 |

*Note*. Cell entries of citations are final-entry unstandardized beta (*b*) coefficients. Coefficients effects accounted for robust standard errors based on bootstrapping to 1,000 resamples with biased corrected confidence set at 95% to assess statistical significance. Bootstrapped standard errors in brackets. * *p* < .05, ** *p* < .01, *** *p* < .001

**Table A3.** *Bootstrapped* *OLS regression testing the association between author gender and h-index in the pooled sample and continent-level without citations*

|  | Pooled sample | Continent-level, *b* (SE) | | | | | |
| --- | --- | --- | --- | --- | --- | --- | --- |
| *Block 1: Scientometric controls* |  | Africa | Asia | Europe | North America | South America | Oceania |
| Research productivity | 0.05*** (0.00) | 0.06*** (0.01) | 0.03*** (0.00) | 0.06*** (0.00) | 0.07*** (0.00) | 0.04*** (0.00) | 0.06*** (0.00) |
| Research experience | 0.76*** (0.02) | 0.19* (0.10) | 0.32*** (0.05) | 0.60*** (0.03) | 0.63*** (0.03) | 0.37** (0.13) | 0.21* (0.09) |
| *ΔR^2^* | 50.9% | 46.1% | 37.2% | 57.1% | 57.9% | 55.5% | 52.4% |
| Block 2: Variable of interest |  |  |  |  |  |  |  |
| Gender_(female)_ | -2.07*** (0.33) | 5.61** (1.84) | -3.24*** (0.82) | -1.85*** (0.50) | -2.92*** (0.58) | -2.49 (1.90) | -4.97** (1.44) |
| ΔR^2^ | 0.1% | 0.8% | 0.2% | 0.1% | 0.1% | 0.3% | 0.5% |
| Total R^2^ | 51% | 46.9 | 37.4% | 57.2% | 58% | 55.8% | 52.9% |
| Adjs. R^2^ | 50.9% | 46.5% | 37.4% | 57.2% | 58% | 55.2% | 52.7% |
| Residual Std. Error | 22.54 | 17.53 | 21.60 | 20.40 | 22.07 | 13.88 | 20.51 |
| N | 21509 | 401 | 3805 | 7920 | 7240 | 236 | 838 |

*Note*. Cell entries of h-index are final-entry unstandardized beta (*b*) coefficients. Coefficients effects accounted for robust standard errors based on bootstrapping to 1,000 resamples with biased corrected confidence set at 95% to assess statistical significance. Bootstrapped standard errors in brackets. * *p* < .05, ** *p* < .01, *** *p* < .001

**Table A4.** *Bootstrapped* *OLS regression testing the association between author gender and h-index at field-level without citations*

|  | Field-level, *b* (SE) | | | | | |
| --- | --- | --- | --- | --- | --- | --- |
| *Block 1: Scientometric controls* | Arts & Humanities | Social sciences | Natural sciences | Medicine | Engineering | Exact sciences & Physics |
| Research productivity | 0.05*** (0.00) | 0.06*** (0.00) | 0.04*** (0.00) | 0.05*** (0.00) | 0.05*** (0.00) | 0.07*** (0.00) |
| Research experience | 0.50*** (0.05) | 0.78*** (0.04) | 0.87*** (0.03) | 1.04*** (0.04) | 0.69*** (0.05) | 0.47*** (0.05) |
| *ΔR^2^* | 47.3% | 51.4% | 39.2% | 56.8% | 52.6% | 56.8% |
| Block 2: Variable of interest |  |  |  |  |  |  |
| Gender_(female)_ | -5.02*** (0.98) | -1.120 (0.66) | -2.22** (0.66) | -1.42 (0.75) | -1.80 (0.94) | -2.44 (1.22) |
| ΔR^2^ | 0.8% | 0% | 0.1% | 0% | 0% | 0.1% |
| Total R^2^ | 48.1% | 51.5% | 39.3% | 56.8% | 52.7% | 56.9% |
| Adjs. R^2^ | 47.9% | 51.4% | 39.3% | 56.8% | 52.6% | 56.8% |
| Residual Std. Error | 18.06 | 21.54 | 21.55 | 23.09 | 19.77 | 24.51 |
| N | 1471 | 4315 | 5576 | 4524 | 2758 | 2865 |

*Note*. Cell entries of h-index are final-entry unstandardized beta (*b*) coefficients. Coefficients effects accounted for robust standard errors based on bootstrapping to 1,000 resamples with biased corrected confidence set at 95% to assess statistical significance. Bootstrapped standard errors in brackets. * *p* < .05, ** *p* < .01, *** *p* < .001.
